# Retinal dystrophy in an individual carrying a de novo missense variant of *SMARCA4*

**DOI:** 10.1002/mgg3.682

**Published:** 2019-04-11

**Authors:** Gerarda Cappuccio, Raffaella Brunetti‐Pierri, Annalaura Torella, Michele Pinelli, Raffaele Castello, Giorgio Casari, Vincenzo Nigro, Sandro Banfi, Francesca Simonelli, TUDP, Nicola Brunetti‐Pierri

**Affiliations:** ^1^ Department of Translational Medicine Federico II University Naples Italy; ^2^ Telethon Institute of Genetics and Medicine Pozzuoli Italy; ^3^ Eye Clinic, Multidisciplinary Department of Medical, Surgical and Dental Sciences University of Campania “Luigi Vanvitelli” Naples Italy; ^4^ Department of Precision Medicine University of Campania “Luigi Vanvitelli” Naples Italy

**Keywords:** Coffin–Siris syndrome, retinitis pigmentosa, *SMARCA4*

## Abstract

**Background:**

Coffin–Siris syndrome (CSS) is characterized by intellectual disability, dysmorphic facial features, growth deficiency, microcephaly, and abnormalities of the fifth fingers/toes. CSS is caused by mutations in several genes of the BRG1‐associated factor pathway including *SMARCA4.*

**Methods:**

Whole‐exome sequencing was performed on a 14‐year‐old female individual who presented with mild intellectual disability and dysmorphic features, tooth abnormalities, and short stature. She had brachydactyly but no aplasia or hypoplasia of the distal phalanx or nail of the fifth digit. She was also found to have retinal dystrophy that has not been previously reported in CSS.

**Results:**

The individual presented herein was found to harbor a previously unreported de novo variant in *SMARCA4.*

**Conclusion:**

This case expands the phenotypic spectrum of CSS manifestations.

## 1 INTRODUCTION

Coffin–Siris syndrome (CSS; CSS1 MIM#135900; CSS2 MIM#614607; CSS3 MIM#614608; CSS4 MIM#614609; CSS5 MIM#616938; CSS6 MIM#617808; CSS7 MIM#618027) is characterized by a broad spectrum of clinical abnormalities including aplasia or hypoplasia of the distal phalanx or nail of the fifth and additional digits, developmental or cognitive delay of varying degree, distinctive facial features, hirsutism/hypertrichosis and sparse scalp hair, growth deficiency, microcephaly, feeding difficulties, and recurrent infections (Kosho, Miyake, & Carey, 2014; Kosho, Okamoto, & Coffin‐Siris Syndrome International, 2014; Wieczorek et al., 2013). Additionally, malformations of central nervous system, heart, gastrointestinal and genitourinary systems have been reported (Vergano & Deardorff, 2014).

CSS is caused by mutations in genes of the human BRG1‐associated factor (BAF) chromatin‐remodeling complex (also known as the SWI/SNF‐A complex) that includes* ARID1A* (MIM#603024), *ARID1B* (MIM#614556), *SMARCA4* (MIM#603254), *SMARCB1* (MIM#601607), and *SMARCE1 *(MIM#603111). Furthermore, variants in *SOX11* (MIM#600898), a downstream transcriptional factor of the BAF complex, and *DPF2* (MIM#601671) encoding a subunit of the BAF complex have been reported (Tsurusaki, Koshimizu, et al., 2014; Vasileiou et al., 2018). *SMARCA4* variants are mostly missense and are localized within the three central domains of the protein Helicase/SANT‐associated, DEAD‐like helicase and Helicase C‐terminal domains (Bramswig et al., 2015; Errichiello et al., 2017; Kosho, Okamoto et al., 2014; Tsurusaki, Okamoto, et al., 2014; Tzeng, du Souich, Cheung, & Boerkoel, 2014). Here, we report an individual harboring a de novo missense *SMARCA4* variant falling outside the central domains who presented with mild intellectual disability, mild and not distinctive dysmorphic features, short stature, tooth agenesis, and retinal dystrophy. This case expands the spectrum of phenotypic abnormalities of CSS.

## 2 CASE REPORT

The child was born to non‐consanguineous parents by caesarean section after 39 weeks of gestation with a birth weight of 2.700 g. She was able to walk independently at 13 months; by 18 months she could say a few words, and she was toilet‐trained at 3 years of age. She had learning difficulties and at the time of the last evaluation, when she was attending third year of high school, she could read and write but she had difficulties with simple calculations. A WISC‐III testing performed when she was 10 years old revealed an IQ of 59. She has good social interactions and had no history of seizures.

**Figure 1:**
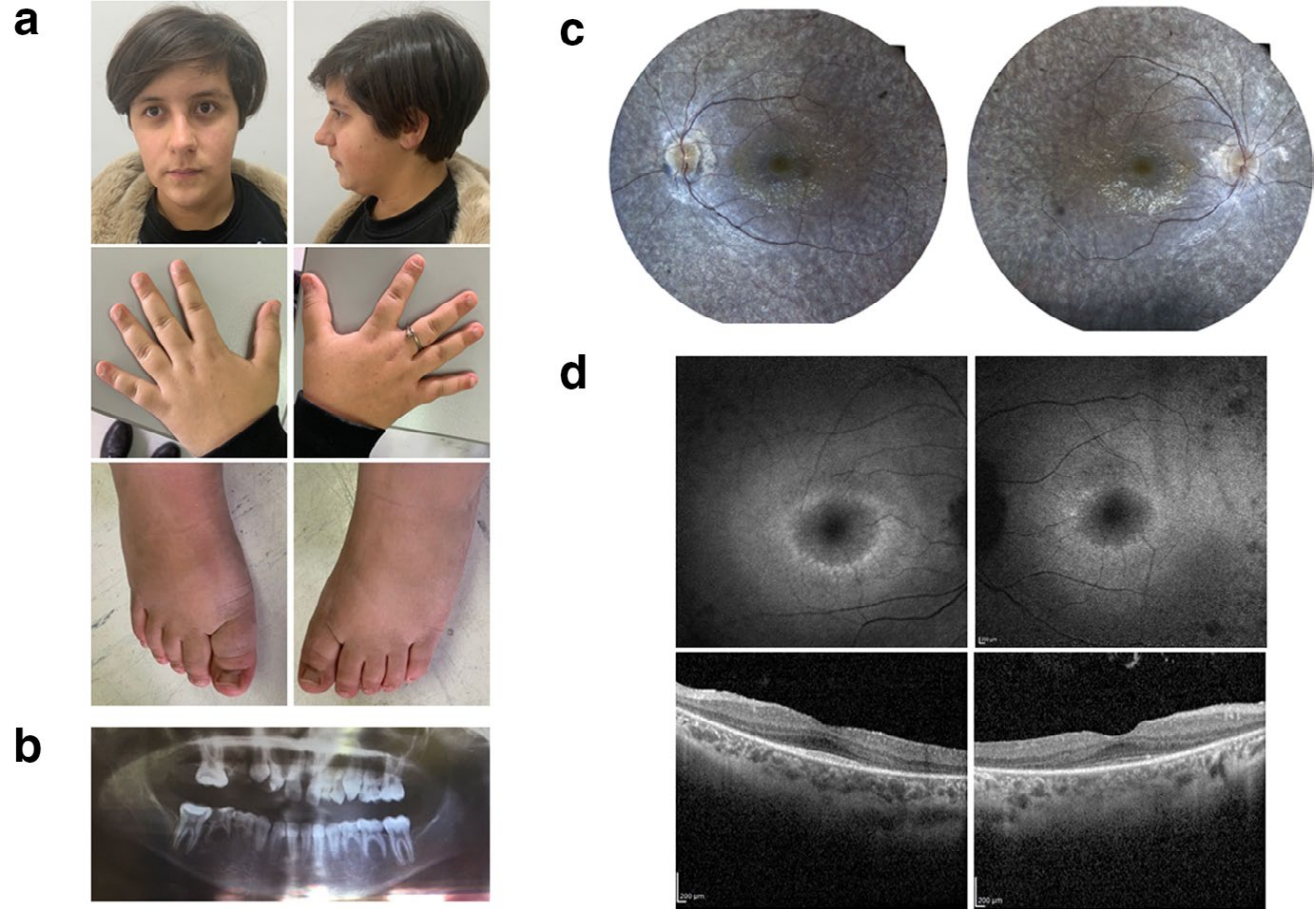
(a) Mild dysmorphic features of the individual herein presented at the age of 14 years and 5 months. The hands showed brachydactyly without aplasia or hypoplasia of the distal phalanx or nail of the fifth digit. Nail hypoplasia of the feet was not observed. (b) X‐rays showing multiple dental anomalies such as small, irregular and absent teeth. (c) Pink optic disc, widespread retinal pigment epithelium dystrophy with pigment deposits in mid‐periphery at retinography. (d) Ring of macular hyper‐autofluorescence at fundus autofluorescence; retinal pigment epithelium dystrophy with vitreo–retinal interface syndrome at optical coherence tomography.

At the age of 14 years and 5 months, her weight was 58 kg (75^th^ centile), height 148 cm (2^nd^ centile) and occipitofrontal circumference 55 cm (55^th^ centile). She showed mild facial dysmorphic features with bulbous nasal tip and micrognathia, and brachydactyly without hypoplasia of the distal phalanx or hypoplastic nails of hands or feet (Figure 1a). She had tooth abnormalities and agenesis confirmed by X‐rays (Figure 1b), and she never underwent dental extraction procedures. She had normal sweating.

By the age of 9 years, she was noted to have night‐blindness and at the age of 14 years, she was found to have a best‐corrected visual acuity of 20/40 in both eyes (right eye [RE], cylinder −1 alpha [axis] 100° left eye [LE], sphere + 1.25 = cylinder −2.25 alpha 90°), abnormal color vision, and normal ocular motility. Lenses were clear. Fundus examination revealed a pink optic disc, widespread dystrophy of retinal pigment epithelium (RPE) with pigment deposits resembling bone spicules in mid‐periphery. Retinal vasculature was normal. Fundus autofluorescence showed a ring of macular hyper‐autofluorescence (Figure 1c). Optical coherence tomography (OCT) performed with the spectral domain OCT (Cirrus HD‐OCT; Carl Zeiss, Dublin, CA) showed RPE dystrophy with vitreo–retinal interface syndrome (Figure 1d). Electroretinography revealed scotopic and photopic traces below noise level.

Auditory brain steam response and EEG did not detect any abnormalities. Brain MRI performed when she was 10‐years old only showed an arachnoid cyst at the ponto–cerebellum junction. The echocardiogram revealed a mild pulmonary insufficiency while abdomen ultrasound was normal.

Chromosome microarray analysis did not detect pathogenic chromosomal rearrangements and Next Generation Sequencing (NGS) and MLPA of a panel of genes responsible for ectodermal dysplasia was negative. NGS of 137 genes responsible for inherited retinal dystrophies also did not detect pathogenic variants. Following informed consent, the proband was enrolled in the Telethon Undiagnosed Diseases Program and genomic DNA from the proband and both her parents underwent whole‐exome sequencing (WES). Genomic DNA was enriched using SureSelect Clinical Research Exome (Agilent, Technologies, Santa Clara, CA) and sequenced with the NextSeq500 sequencing system (Illumina, San Diego, CA). A custom pipeline based on Burrows–Wheeler Alignment tool (BWA) Genome Analysis Toolkit (GATK), and ANNOVAR (Wang, Li, & Hakonarson, 2010) were used to call, annotate, filter, and prioritize variants. WES identified in the proband a de novo heterozygous variant c.4297G >A in exon 31 of *SMARCA4* resulting in the p.(Glu1433Lys) amino acid change (NM_001128849, chr19: g.11,152,013G > A). The variant was confirmed by Sanger sequencing in the proband and was found to be absent in both parents. The affected amino acid residue: (a) is located between the Helicase C‐Terminal domain and the Bromodomain, (b) it affects an evolutionarily highly conserved residue, (c) is not reported neither in ExAc nor in GnomAD, (d) is predicted to be pathogenic by Polyphen, and (e) has a CADD score of 28.8. Taken together and based on current guidelines (Richards et al., 2015), this *SMARCA4* variant is classified as likely pathogenic.

## 3 DISCUSSION

Components of BAF complex modulate gene expression and cell differentiation via nucleosome remodeling. Therefore, loss of functional BAF complex can affect gene expression resulting in pleiotropic phenotypic manifestations (Kosho et al., 2014). The individual herein reported presented with a mild CSS phenotype and showed retinal dystrophy that has not been previously reported in CSS. She had brachydactyly but no aplasia or hypoplasia of the distal phalanx or nail of the fifth digit. Moreover, her dysmorphic features were mild and non‐specific. Minor dental anomalies such as small, conic, pointed, widely spaced teeth along with delayed dentition have been reported in CSS individuals (Hoyer et al., 2012; Wieczorek et al., 2013) including subjects carrying *SMARCA4* variants (Errichiello et al., 2017). Therefore, tooth abnormalities we observed in our case are part of the spectrum of dental anomalies of CSS. Noteworthy, variants in genes responsible for oligodontia and ectodermal dysplasia (e.g., *PAX9, EDA, MSX1, AXIN2, EDARADD, NEMO,* and* KRT17*) (Ye & Attaie, 2016) were not detected by either the targeted sequencing or WES.

Eye abnormalities have been reported in CSS individuals including subjects with *SMARCA4* variants, and they include severe myopia, strabismus, microphthalmia, and spherophakia (Errichiello et al., 2017; Kosho et al., 2014). However, retinal pigmentary defects were not previously reported neither in individuals harboring *SMARCA4* variants nor other genes of the BAF complex. Both targeted sequencing of genes responsible for inherited retinopathies and WES ruled out inherited retinal disorders in our case. Interestingly, the retinal defect detected in the individual herein described is paralleled by a disorganized retinal structure, abnormal retinal lamination, and disrupted RPE pigmentation observed in *smarca4* null zebrafish model (Zhang et al., 2014; Zhang, Bonilla, Chong, & Leung, 2013), suggesting that retinal dystrophy is indeed due to *SMARCA4* defects.

*SMARCA4* missense variants in CSS individuals are largely clustered within the three central domains of the protein (Helicase/SANT‐associated, DEAD‐like helicase and helicase c‐terminal) (Tsurusaki, Okamoto, et al., 2014), whereas the variant detected in our case did not affect any of the central domains and is among the most C‐terminal missense variants reported so far. The location of the variant might explain the atypical phenotype of our case presenting with retinopathy but also without the typical dysmorphic features and digital abnormalities of CSS. Nevertheless, further individuals harboring *SMARCA4* variants are needed to confirm whether retinal abnormalities are dependent on *SMARCA4* defects. Moreover, further studies are needed to evaluate whether retinal changes are specific for *SMARCA4* defects or might be due also to the defects of other genes of the BAF complex causing CSS.

## CONFLICT OF INTEREST

The authors declare no conflict of interest.
